
JOURNAL INFORMATION
==============================
NLM Title Abbreviation: Biospektrum (Heidelb)
Journal ID: Biospektrum (Heidelb)
NLM Journal ID: 9615685

Biospektrum

ISSN: 0947-0867
EISSN: 1868-6249

ARTICLE INFORMATION
==============================
PMCID: PMC10101533
PMID: 37073321
DOI: 10.1007/s12268-023-1900-4
Article ID: 1900
Article version: 1
Subjects: Wissenschaft · Special: Molekulare Medizin & Virologie

30 Jahre Nanobodies: Neues von kleinen Helfern mit großem Potenzial

Wagner Teresa R. 1 2
Burgstaller Sandra 1 3
Frecot Desiree I. 1 2
Lukowski Robert 3
Rothbauer Ulrich ulrich.rothbauer@uni-tuebingen.de
1 2
1 grid.10392.39 0000 0001 2190 1447 Pharmazeutische Biotechnologie, Universität Tübingen, Tübingen, Deutschland
2 grid.461765.7 0000 0000 9457 1306 NMI Naturwissenschaftliches und Medizinisches Institut an der Universität Tübingen Pharmazeutische Biotechnologie, Markwiesenstraße 55, D-72770 Reutlingen, Deutschland
3 grid.10392.39 0000 0001 2190 1447 Pharmakologie, Toxikologie und Klinische Pharmazie, Universität Tübingen, Tübingen, Deutschland
Electronic publication date: 2023 Apr 13
Print publication date: 2023
Volume: 29
Issue: 2
First page: 145
Last page: 149
Copyright: © Die Autorinnen und Autoren 2023
License: Open Access: Dieser Artikel wird unter der Creative Commons Namensnennung 4.0 International Lizenz veröffentlicht, welche die Nutzung, Vervielfältigung, Bearbeitung, Verbreitung und Wiedergabe in jeglichem Medium und Format erlaubt, sofern Sie den/die ursprünglichen Autor(en) und die Quelle ordnungsgemäß nennen, einen Link zur Creative Commons Lizenz beifügen und angeben, ob Änderungen vorgenommen wurden. Die in diesem Artikel enthaltenen Bilder und sonstiges Drittmaterial unterliegen ebenfalls der genannten Creative Commons Lizenz, sofern sich aus der Abbildungslegende nichts anderes ergibt. Sofern das betreffende Material nicht unter der genannten Creative Commons Lizenz steht und die betreffende Handlung nicht nach gesetzlichen Vorschriften erlaubt ist, ist für die oben aufgeführten Weiterverwendungen des Materials die Einwilligung des jeweiligen Rechteinhabers einzuholen. Weitere Details zur Lizenz entnehmen Sie bitte der Lizenzinformation auf http://creativecommons.org/licenses/by/4.0/deed.de.
License URL: https://creativecommons.org/licenses/by/4.0/

issue-copyright-statement: © Springer-Verlag GmbH Deutschland, ein Teil von Springer Nature 2023

==============================
2023 marks the 30th anniversary of the discovery of single-domain antibody fragments in camelids, better known as nanobodies. This was the starting point for their tremendous success story in biomedicine. Here we highlight recent advances in the development of nanobodies for the detection of neutralizing SARS-CoV-2 antibodies, as biosensors for monitoring extracellular metabolites and as tracer molecules for non-invasive imaging of immune cells.

■ 2023 feiern wir den 30. Jahrestag der Entdeckung der Schwere-Ketten-Antikörper (*heavy-chain-only antibodies*) aus Kameliden und damit den Beginn der biomedizinischen Erfolgsgeschichte der Einzeldomänen-Antikörpern, den Nanobodies (Nbs) [1]. Im Vergleich zu konventionellen Antikörpern vom Immunglobulin-G(IgG)-Typ, haben Nbs einzigartige Vorteile. Mit einem Molekulargewicht von ~15 kDa bei einer Größe von ca. 2 × 4 nm sind sie nicht nur 10-fach kleiner als IgGs, sondern zeichnen sich auch durch hohe Löslichkeit, thermische und chemische Stabilität, kostengünstige Produktion, hohe Gewebepenetration und geringe Immunogenität aus. Gleichzeitig lassen sie sich effektiv und flexibel sowohl chemisch als auch genetisch funktionalisieren [2]. Aufgrund dieser Eigenschaften werden Nbs zunehmend in der biomedizinischen Forschung sowie in der Diagnostik und Therapie in vielfältigen Formaten eingesetzt. Im Folgenden stellen wir drei Anwendungsmöglichkeiten von Nbs als Antikörpersurrogate, Biosensor-Fusionskonstrukte und PET-Tracer als Beispiele für das große Potenzial dieser Bindemoleküle vor.

## SARS-CoV-2 – Überwachung des Immunstatus mittels Nanobodies

Mit dem Ausbruch der COVID-19-Pandemie wurden schnell verfügbare Diagnoseverfahren zur Abschätzung des Infektionsausmaßes sowie des Schutzes durch neu entwickelte Impfungen in der Bevölkerung benötigt. Studien haben schon früh gezeigt, dass ein Immunschutz durch neutralisierende Antikörper vermittelt wird, welche die rezeptorbindende Domäne (RBD) blockieren, mit der das Spike-Protein des SARS-CoV-2-Virus an das Angiotensin-Converting-Enzym 2

Hier steht eine Anzeige.

Springer

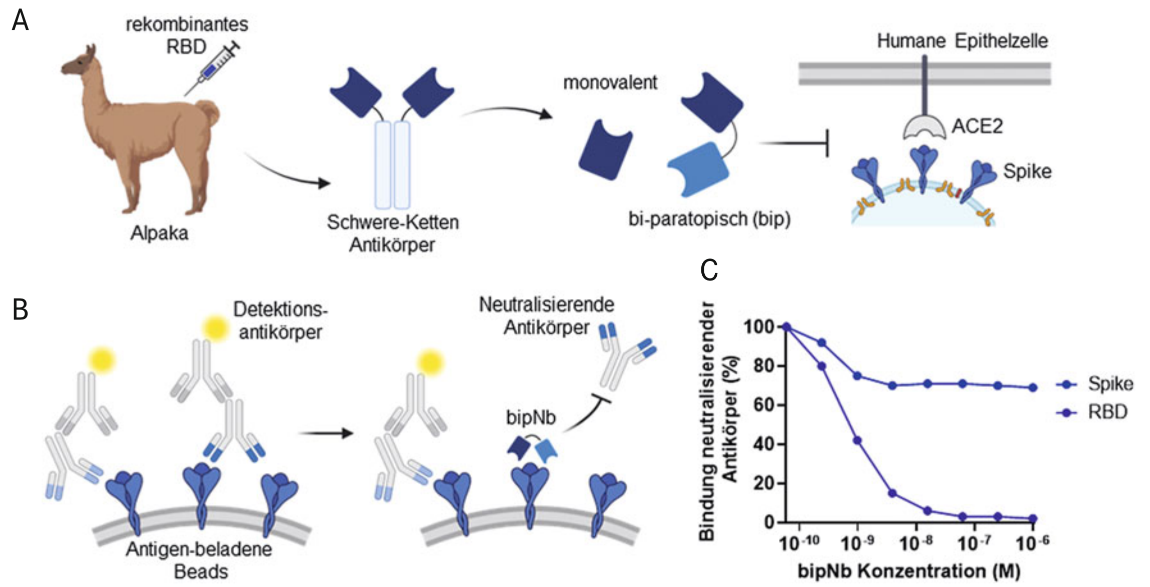

▲ **Abb. 1:** NeutrobodyPlex-Prinzip. **A,** Zur Entwicklung von Nanobodies (Nbs), die spezifisch die rezeptorbindende Domäne (RBD) des Spike-Proteins von SARS-CoV-2 erkennen, wurden aus einem immunisierten Alpaka verschiedene variable Domänen von Schwere-Ketten-Antikörpern gentechnisch isoliert. Die Interaktion zwischen dem Spike-Protein und dem Angiotensin-Converting-Enzym 2 (ACE) wird durch RBD-spezifische Nbs im monovalenten oder bi-paratopischen Format blockiert. **B,** NeutrobodyPlex: Zur Bindung von Antikörpern werden SARS-CoV-2-Antigen-gekoppelte Beads mit Patientenserum inkubiert. Durch Zugabe von bipNbs werden spezifisch die neutralisierenden Antikörper verdrängt. **C,** Die Abnahme des Signals auf RBD mit zunehmender bipNb-Konzentration entspricht der Menge an neutralisierenden Antikörper im Serum, während das Signal auf Spike die gesamte Antikörperantwort widerspiegelt. Erstellt mit BioRender.com.

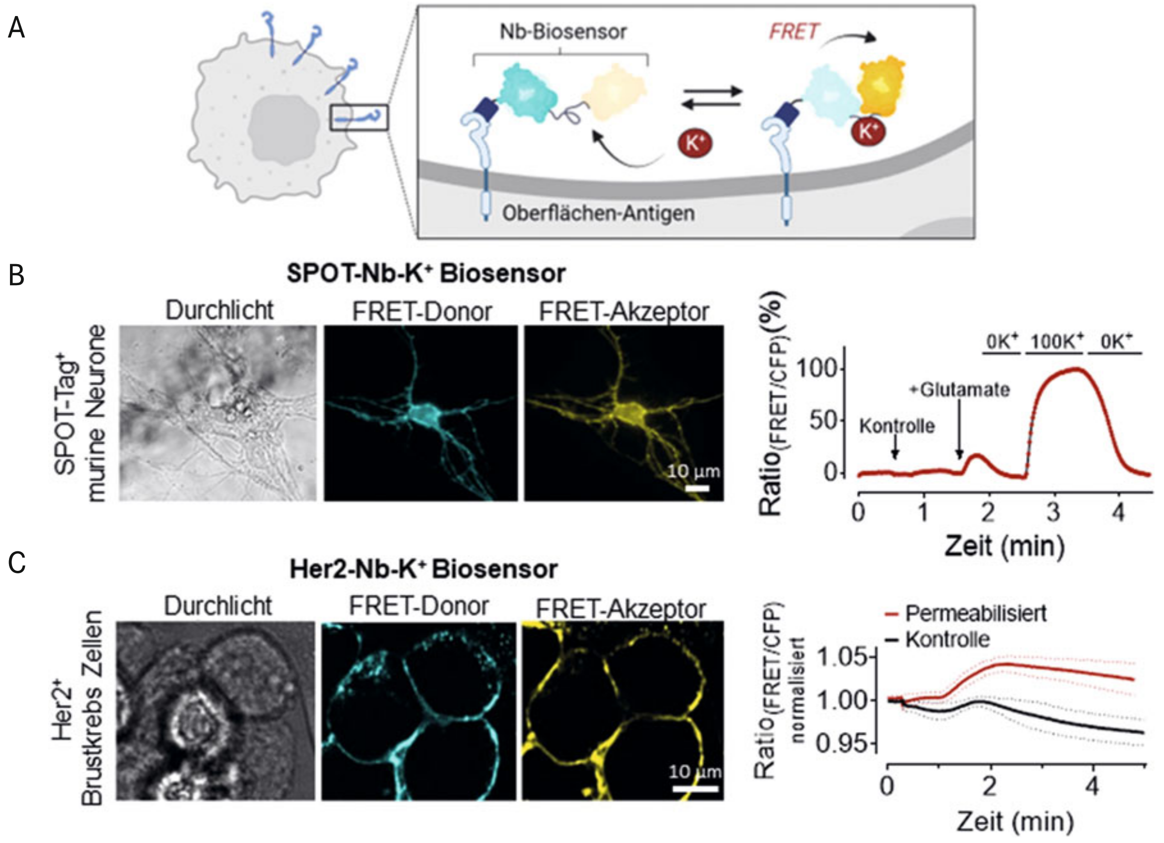

▲ **Abb. 2:** Nanobody-fusionierte Biosensoren. **A,** FRET-basierte Biosensoren werden über Nb/Antigen-Interaktion spezifisch auf Zelloberflächen immobilisiert. Dies ermöglicht eine räumlich aufgelöste Quantifizierung von Veränderungen extrazellulärer Ionen und Metaboliten, z. B. $K^+$. **B,** Lebendfluoreszenzfärbung von SPOT-Tag$^+$ murinen Neuronen mit SPOT-Nb-$K^+$-Biosensor und quantitative Analyse des $K^+$-spezifischen FRET-Signals nach Glutamat- bzw. $K^+$-Zugabe. **C,** Lebendfluoreszenzfärbung von Her2$^+$-Zellen mit Her2-Nb-$K^+$-Biosensor und $K^+$-spezifische FRET-Ratio nach Permeabilisierung. Erstellt mit BioRender.com.

(ACE2) bindet, um Zellen zu infizieren. Die schnell entwickelten serologischen SARS-CoV-2-Antikörpertests konnten allerdings nicht zwischen neutralisierenden und nicht neutralisierenden Antikörpern unterscheiden. Um die Neutralisationskapazität von Serumproben zu bestimmen, wurden zeit- und arbeitsaufwendige Virusneutralisationstests durchgeführt. Unser Ziel war es daher, Nbs als Antikörpersurrogate zu entwickeln, mit denen sich neutralisierende Antikörper in Serumproben im Hochdurchsatzverfahren nachweisen lassen. Dazu haben wir im ersten Schritt aus einer Nb-Genbibliothek eines mit der RBD von SARS-CoV-2-immunisierten Alpakas eine Vielzahl an Nbs selektiert und durch detaillierte Epitop-Kartierungen und Kristallstrukturanalysen Kandidaten identifiziert, die unterschiedliche Bereiche der RBD binden und funktionell eine Interaktion der RBD mit ACE2 inhibieren (**Abb. 1A**). Im zweiten Schritt wurden die zwei wirksamsten Nb-Kandidaten mit unterschiedlichen Bindeepitopen zu einem bi-paratopischen (bip) Nb fusioniert. Damit konnten wir nicht nur eine synergistische Inhibition der RBD/ACE2-Interaktion erzielen, sondern auch die Bindungseigenschaften der Nbs für mutierte Versionen der RBD, wie sie in kontinuierlich neuen SARS-CoV-2-Varianten entdeckt wurden, sicherstellen. Um abschließend neutralisierende Antikörper in Serumproben nachzuweisen, wurden die bipNbs als Antikörpersurrogate in einem kompetitiven Bindungstest, dem NeutrobodyPlex, eingesetzt (**Abb. 1B**, [3]). Durch die gezielte Verdrängung der neutralisierenden Serumantikörper durch anti-RBD-bipNbs ließ sich die Neutralisierungskapazität von Serumproben von infizierten oder geimpften Personen quantitativ bestimmen (**Abb. 1C**). Diese indirekte Messung der Antikörperspiegel lieferte nach Automatisierung innerhalb weniger Stunden Ergebnisse bei einer Vielzahl an Proben. In Summe ist es mithilfe des NeutrobodyPlex nun möglich, den Immunstatus großer Populationen kontinuierlich zu überwachen und beispielsweise den Erfolg von Impfkampagnen in der Bevölkerung zu beurteilen [3].

## Nanobody-fusionierte Biosensoren: quantitative Detektion extrazellulärer Ionen und Metabolite in Echtzeit

Mit FRET-(Förster-Resonanz-Energietransfer)basierten Biosensoren lassen sich optisch Veränderungen extrazellulärer Ionen und Metabolite in biologischen Systemen quantitativ bestimmen. Eine bis dato ungelöste Herausforderung liegt jedoch darin, diese Änderungen bestimmten Zellen bzw. Geweben zuzuordnen und ortsaufgelöst zu messen. Vor diesem Hintergrund bieten Nbs als kleine und biotechnologisch einfach zu manipulierende Bindemoleküle neue Möglichkeiten, Biosensoren spezifisch an Zielgewebe zu immobilisieren. Um dieses Potenzial zu testen, wurden Nbs genetisch an bekannte fluoreszierende FRET-Biosensoren gekoppelt, die auf $K^+$-, pH- und Glukoseänderungen reagieren [4–6], und deren Bindungs- und Funktionseigenschaften im Detail an lebenden Zellen untersucht (**Abb. 2A**, [7]).

Am Beispiel eines Nbs (SPOT-Nb) der ein generisches Peptid-Epitop (SPOT-Tag)

Hier steht eine Anzeige.

Springer

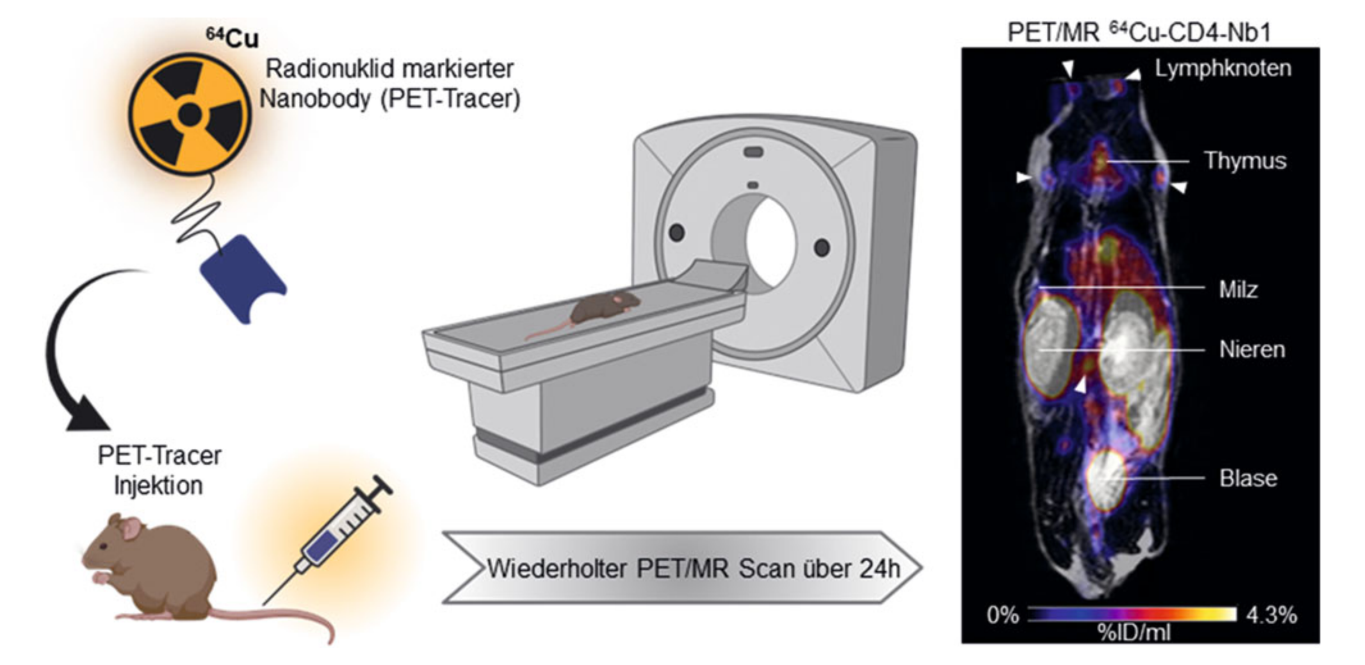

▲ **Abb. 3:** Nicht invasive Bildgebung mit einem CD4-spezifischen Nanobody. Zum Nachweis der $CD4^+$-T-Zellen wurde ein Radionuklid markierter CD4-spezifischer Nb ($^{64}$Cu-CD4-Nb) generiert. PET/MR-Scans von Mäusen, die das humane CD4-Molekül exprimieren, zeigen eine spezifische Anreicherung in Geweben mit hohem Anteil an $CD4^+$-T-Zellen inklusive Lymphknoten (weiße Pfeile), Thymus und Milz. Erstellt mit BioRender.com.

bindet, konnten wir demonstrieren, dass Nb-fusionierte Biosensoren sich zielgerichtet und effektiv auf Oberflächen verschiedener Zelltypen, z. B. primären Neuronen, immobilisieren lassen (**Abb. 2B**). Gleichzeitig wurde durch Protein Engineering die notwendige Mobilität der FRET-Donor- bzw. Akzeptor-Einheiten und damit die Funktionalität der Sensoren beibehalten. Mit dem SPOT-Nb-$K^+$-Biosensor war es so z. B. möglich, die neuronale Freisetzung von $K^+$ nach rezeptorspezifischer Stimulation mit Glutamat zeit- und ortsaufgelöst auf SPOT-Tag-positiven (SPOT-$Tag^+$) Mausneuronen darzustellen (**Abb. 2B**). Zur Adressierung eines endogenen Zielmoleküls haben wir Biosensoren mit einem für den humanen epidermalen Wachstumsfaktor-Rezeptor Typ 2 (Her2) spezifischen  kombiniert. Auch damit ließen sich u. a. Änderungen der extrazellulären Kaliumkonzentration nach gezielter Permeabilisierung auf Her2-positiven ($Her2^+$) Brustkrebszellen zeit- und ortaufgelöst quantitativ bestimmen (**Abb. 2C**). Grundsätzlich eigneten sich alle generierten Nb-fusionierten Biosensoren aufgrund ihrer hohen Affinität, geringen Dissoziation und moderaten Endozytose zur optischen Langzeitmessung von Ionen- und Metabolitänderungen in lebenden Systemen. Mit dem flexibel einsetzbaren SPOT-Nb/SPOT-Tag-System stehen nun diverse Biosensoren zur Verfügung, mit denen sich die extrazelluläre Dynamik von Analyten in Zellsystemen untersuchen lässt, für die es noch keine spezifischen Nbs gibt. Gleichzeitig liefern die Her2-Nb-fusionierten Biosensoren neue Erkenntnisse zur Rolle extrazellulärer Ionen und Metabolite in Tumormodellen. Zukünftig können Nb-gekoppelte Biosensoren einen erheblichen Beitrag dazu leisten, den Effekt von genetischen oder pharmakologischen Interventionen auf das extrazelluläre Milieu sichtbar zu machen. Diese Ansätze werden zu einem besseren Verständnis der Wechselwirkung des Tumormilieus auf Krebszellen und umliegende Zellen beitragen. Letztlich könnten Nb-gekoppelte Biosensoren suspekte Veränderungen in Gewebe- oder Zellproben aufzeigen und bei der Entdeckung sowie Entwicklung neuer Therapieansätze helfen.

## Nanobodies zur nicht invasiven Bildgebung von Immunzellen

Angesichts des therapeutischen Fortschritts im Bereich immunassoziierter Erkrankungen, z. B. Autoimmunerkrankungen, viraler Infektionen und Krebs, wächst der Bedarf an diagnostischen Verfahren zur personalisierten Auswahl einer geeigneten Behandlungsstrategie. Aktuelle diagnostische Verfahren basieren mehrheitlich auf invasiver Probenentnahme und Endpunktanalysen, was ihre Durchführung und Aussagekraft z. T. stark limitiert. Nicht invasive bildgebende Verfahren – wie die Positronenemissionstomografie (PET) – bieten hier eine vielversprechende Alternative. Hierbei werden mit radioaktiv markierten Bindemolekülen, den Tracern, therapeutisch relevante Marker und biologische Prozesse im Organismus visualisiert. Aufgrund ihrer hohen Spezifität, schnellen Gewebspenetration und kurzen Verweildauer im Organismus sind insbesondere Nbs für die Entwicklung solcher PET-Tracer attraktiv [8]. Diese Eigenschaften nutzend, haben wir in Kooperation mit dem Werner Siemens Imaging Center in Tübingen kürzlich CD4-spezifische Nbs entwickelt. Auf Basis biochemischer, struktureller und biologischer Charakterisierungen konnten wir Kandidaten mit hoher Affinität und Spezifität für humanes CD4 identifizieren. Für einen ersten Leitkandidaten CD4-Nb1 wurde das erkannte Epitop im Detail bestimmt und eine Beeinträchtigung der T-Zell-Funktion nach Bindung durch *in vitro*-Analysen ausgeschlossen. Zur Generierung eines PET-Tracers wurde der CD4-Nb1 mit dem Isotop $^{64}$Cu radioaktiv markiert ($^{64}$Cu-CD4-Nb1). Bildgebungsdaten aus einem humanisierten CD4-Mausmodell zeigen eine spezifische Akkumulierung des Tracer-Signals in $CD4^+$-T-Zell reichen Organen, wie Thymus, Milz und den Lymphknoten (**Abb. 3**, [9]). Mit dem neu entwickelten CD4 PET-Tracer werden aktuell in präklinischen Mausmodellen die Infiltration von $CD4^+$-T-Zellen in Tumorläsionen nach Behandlung mit Immuncheckpoint-Inhibitoren bzw. nach Induzieren einer lokalen Kontaktallergie in distinkte Inflammationsherde untersucht. Zusammenfassend ist es mit CD4-Nb-basierten Tracern nun erstmals möglich, die Lokalisation und die Dynamik von $CD4^+$-T-Zellen und damit einer essenziellen Komponente des Immunsystems mittels *in vivo*-Bildgebungsverfahren zu bestimmen. Perspektivisch können CD4-Nbs zur Auswahl geeigneter Krebstherapien und zur Langzeitbeobachtung ihres Verlaufs eingesetzt werden.

## AUTORINNEN UND AUTOREN

**Arbeitsgruppe**

Hier steht eine Anzeige.
Springer


Funding note: Open Access funding enabled and organized by Projekt DEAL.

Arbeitsgruppe Die Brückenprofessur für Pharmazeutische Biotechnologie unter der Leitung von Prof. Dr. Ulrich Rothbauer am NMI Naturwissenschaftliches und Medizinisches Institut an der Universität Tübingen dient als Bindeglied für den Wissenstransfer aus der Grundlagenforschung in die anwen dungs orientierte Forschung und Entwicklung. Im Fokus steht die Entwicklung und Funktionalisierung rekombinanter Antikörper (Nano- und Chromobodies) für biomedizinische Anwendungen wie Proteomik, zelluläre und in vivo-Bild-gebung sowie Therapeutika.

Robert Lukowski, Sandra Burgstaller, Desiree Frecot, Teresa Wagner und Ulrich Rothbauer (v. l. n. r.)


Literatur

[1] Hamers-Casterman C Atarhouch T Muyldermans S Naturally occurring antibodies devoid of light chains Nature 1993 363 446 448 10.1038/363446a0 8502296
[2] Wagner TR Rothbauer U Nanobodies — Little helpers unravelling intracellular signaling Free Radic Biol Med 2021 176 46 61 10.1016/j.freeradbiomed.2021.09.005 34536541
[3] Wagner TR Ostertag E Kaiser PD NeutrobodyPlex-monitoring SARS-CoV-2 neutralizing immune responses using nanobodies EMBO Rep 2021 22 e52325 10.15252/embr.202052325 33904225 PMC8097376
[4] Takanaga H Chaudhuri B Frommer WB GLUT1 and GLUT9 as major contributors to glucose influx in HepG2 cells identified by a high sensitivity intramolecular FRET glucose sensor Biochim Biophys Acta 2008 1778 1091 1099 10.1016/j.bbamem.2007.11.015 18177733 PMC2315637
[5] Bischof H Rehberg M Stryeck S Novel genetically encoded fluorescent probes enable real-time detection of potassium in vitro and in vivo Nat Commun 2017 8 1422 10.1038/s41467-017-01615-z 29127288 PMC5681659
[6] Burgstaller S Bischof H Gensch T pH-Lemon, a Fluorescent Protein-Based pH Reporter for Acidic Compartments ACS Sens 2019 4 883 891 10.1021/acssensors.8b01599 30864782 PMC6488996
[7] Burgstaller S Wagner TR Bischof H Monitoring extracellular ion and metabolite dynamics with recombinant nanobody-fused biosensors iScience 2022 25 104907 10.1016/j.isci.2022.104907 36046190 PMC9421384
[8] Lecocq Q De Vlaeminck Y Hanssens H Theranostics in immuno-oncology using nanobody derivatives Theranostics 2019 9 7772 7791 10.7150/thno.34941 31695800 PMC6831473
[9] Traenkle B Kaiser PD Pezzana S Single-Domain Antibodies for Targeting, Detection, and In Vivo Imaging of Human CD4(+) Cells Front Immunol 2021 12 799910 10.3389/fimmu.2021.799910 34956237 PMC8696186
